# Evidence of host-virus co-evolution in tetranucleotide usage patterns of bacteriophages and eukaryotic viruses

**DOI:** 10.1186/1471-2164-7-8

**Published:** 2006-01-18

**Authors:** David T Pride, Trudy M Wassenaar, Chandrabali Ghose, Martin J Blaser

**Affiliations:** 1 Department of Medicine, Division of Infectious Diseases And Geographic Medicine, Stanford University School of Medicine, Stanford, CA, USA; 2 Molecular Microbiology and Genomics Consultants, Zotzenheim, Germany; 3 Department of Medicine, Division of Infectious Diseases, Harvard Medical School, Boston, MA, USA; 4 Departments of Medicine and Microbiology, New York University School of Medicine and VA Medical Center, New York, NY4, USA

## Abstract

**Background:**

Virus taxonomy is based on morphologic characteristics, as there are no widely used non-phenotypic measures for comparison among virus families. We examined whether there is phylogenetic signal in virus nucleotide usage patterns that can be used to determine ancestral relationships. The well-studied model of tail morphology in bacteriophage classification was used for comparison with nucleotide usage patterns. Tetranucleotide usage deviation (TUD) patterns were chosen since they have previously been shown to contain phylogenetic signal similar to that of 16S rRNA.

**Results:**

We found that bacteriophages have unique TUD patterns, representing genomic signatures that are relatively conserved among those with similar host range. Analysis of TUD-based phylogeny indicates that host influences are important in bacteriophage evolution, and phylogenies containing both phages and their hosts support their co-evolution. TUD-based phylogeny of eukaryotic viruses indicates that they cluster largely based on nucleic acid type and genome size. Similarities between eukaryotic virus phylogenies based on TUD and gene content substantiate the TUD methodology.

**Conclusion:**

Differences between phenotypic and TUD analysis may provide clues to virus ancestry not previously inferred. As such, TUD analysis provides a complementary approach to morphology-based systems in analysis of virus evolution.

## Background

Eukaryotic viruses and bacteriophages exist in numerous forms and are capable of infecting disparate hosts. The taxonomy of viruses is based upon morphological features, including capsid and tail structures, specific type of genetic material, and mechanism of replication and assembly [1,2]. Genetic comparison across virus species has been complicated by generally different rates of gene evolution, thus, their overall classification rests on phenotypic and morphologic characteristics [3]. Horizontal gene transfer has been substantial in virus evolution [4-7], complicating reproduction of ontogeny based on the current presence of particular loci. Analysis of phylogenies based on phenotypic systems is limited by convergent evolution, in which like characteristics are evolved by unrelated organisms to suit particular niches or evolutionary requirements [8]. Taxonomy of bacteriophages also has been based on morphologic characteristics [9,10]. Tail morphology forms the basis for bacteriophage classification into 3 separate families: Myoviridae (contractile tails), Podoviridae (short tail stubs), and Siphoviridae (long tails) [1]. Studies examining phage tail assemblies [11] have not ascertained whether the source of tail characteristics has phylogenetic significance, indicating the likely existence of polyphylogeny within these phage groups [12].

Unlike viruses, prokaryotes most commonly have been taxonomically classified according to a single locus, 16S rRNA [13-15]. Because of its relatively conservative rate of evolution, and presumed rarity of horizontal transfer due to its functional constraints, the 16S rRNA locus is believed to serve as an accurate marker of recent common ancestry [15].

Evaluation of prokaryotic ancestry based on shared gene content also has been proposed, guided by the principle that prokaryotes have cores of essential genes, whose presence or absence is evolutionarily significant [16-18]. Alternatively, there has been analysis of phylogenetic signal in whole-genome nucleotide usage patterns and is consistent with the predicted phylogenetic structure of prokaryotes based on 16S rRNA [19]. Whole-genome approaches are less biased by any single locus [20], with horizontal transfer being an intrinsic part of the signal, reflecting the current. Using Zero-Order Markov algorithms, we have previously demonstrated that patterns of tetranucleotide usage patterns retain phylogenetic signal among many related prokaryotes [19]. Differences between tetranucleotide usage and 16S rRNA in ancestral reconstruction likely are due to horizontal influences, such as extensive recombination, and/or presence of restriction/modification systems.

We sought to better understand the evolution of viruses by comparing methods for reproducing ancestry including tetranucleotide usage deviation, and shared gene content to construct a framework independent of phenotypic analysis. Our goals were to understand whether: 1) phylogenetic signal is retained in nucleotide usage patterns of viruses; 2) phylogenetic structures based on nucleotide usage patterns and tail morphology are similar; 3) nucleotide usage patterns in viruses are primarily determined by gene content; and 4) whether their prokaryotic hosts exert a substantial influence on bacteriophage nucleotide usage patterns.

## Results

### Conservation of tetranucleotide usage patterns across bacteriophage genomes

Nucleotide usage patterns are unique among different prokaryotes, providing distinct signatures [19,21,22] that are well-conserved across each genome, except for DNA hypothesized to be acquired through lateral gene transfer [23,24]. To determine whether bacteriophages have unique genomic signatures, we employed a method based on tetranucleotide usage deviations (TUD) from expected. TUD patterns have substantially more phylogenetic signal than codon usage biases when compared to 16S rRNA [19]. We generated TUD based on Zero-Order Markov algorithms, which determine how patterns of tetranucleotide usage in each genome deviate from those expected based on overall nucleotide content, representing the genomic signature [19,25]. Zero-order Markov algorithms were chosen, as removal of constituent oligonucleotide biases (e.g. dinucleotide and trinucleotide biases) through Markov chain analysis results in a substantial loss of TUD phylogenetic signal [19].

**Figure 1:**
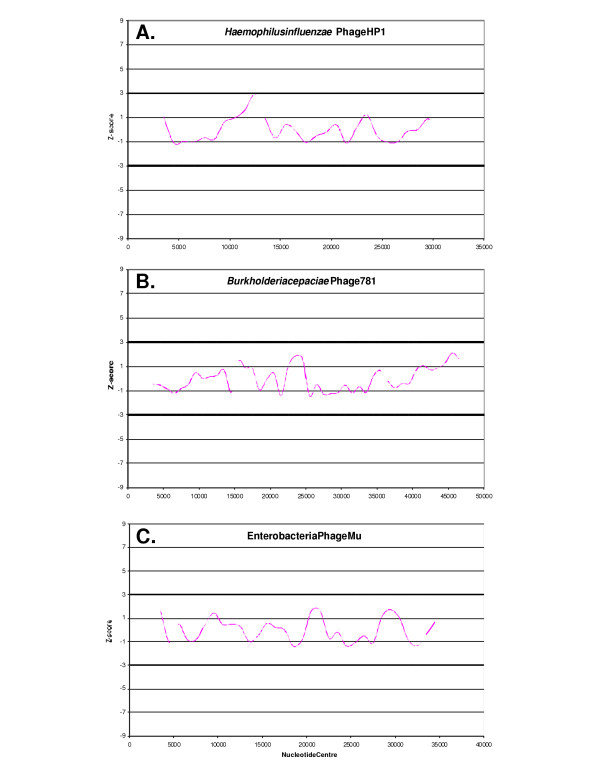
Tetranucleotide difference analysis of representative bacteriophage genomes (*H. influenzae *phage HP1, *B. cepaciae *phage 781, and Enterobacteria phage Mu). Tetranucleotide differences were determined with window and step sizes of 5,000 and 1,000, respectively, and Z-scores were determined as described in Materials and Methods. Solid black lines represent Z-scores of ± 3.

**Figure 2:**
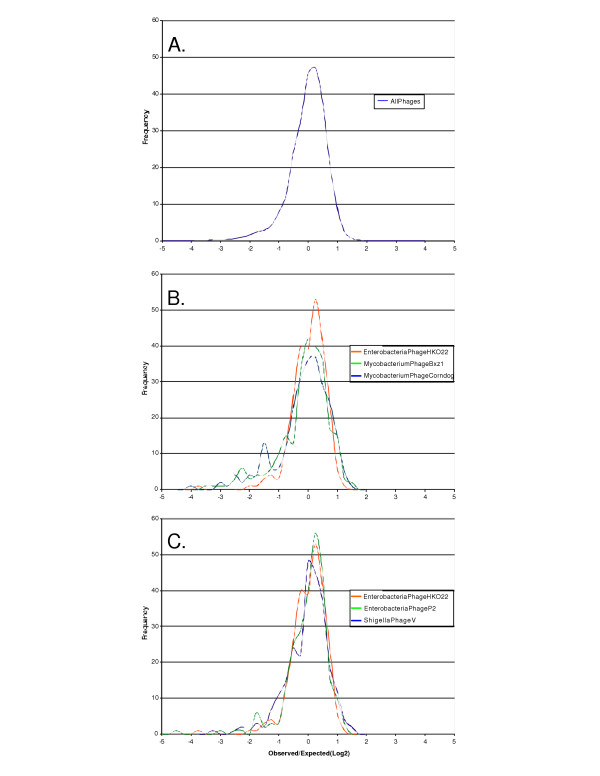
Frequency distribution of DNA tetranucleotide usage profiles of selected bacteriophages. The observed/expected TUD was determined for the 256 tetranucleotide combinations for each genome, as described in Materials and Methods. The resulting values were sorted within 0.25 intervals and the ordinate represents the number of tetranucleotide combinations within each interval. Panel A: Blue – All bacteriophages studied. Panel B: Red – Enterobacteria phage HK022 (Siphoviridae), Green – Enterobacteria phage P2 (Myoviridae), Blue – *Shigella *phage V (Podoviridae). Panel C: Red – Enterobacteria phage HK022, Green – *Mycobacterium *phage Bxz1 (Myoviridae), Blue – *Mycobacterium *phage Corndog (Siphoviridae).

To determine whether TUD patterns are conserved, we measured TUD profiles across representative bacteriophage genomes, comparing each portion tested against the genome mean [24]. The patterns of tetranucleotide usage are relatively well-conserved across all bacteriophages studied [see Additional file 1], with examples provided for *Haemophilus *phage HP1 (Figure 1a), *Burkholderia *phage 781 (Figure 1b), and Enterobacteriaphage Mu (Figure 1c). The relatively small variation in patterns of nucleotide usage across phage genomes indicates that mean genome nucleotide usage patterns can be considered representative as a first-order approximation. TUD profiles of all phage genomes combined follows a normal distribution (Figure 2a), which further supports that tetranucleotide usage patterns are well-conserved across each bacteriophage studied.

We next sought to determine whether the TUD distribution across each bacteriophage genome is unique, representing a genomic signature. Highly similar yet distinct TUD profiles were shown for Enterobacteria phage P2 (Myoviridae), Enterobacteria phage HK022 (Siphoviridae), and *Shigella *phage V (Podoviridae) (Figure 2b). However, within a bacteriophage family, TUD patterns are not strictly conserved, as demonstrated by the lack of similarity between *Mycobacterium *phage Bxz1 and Enterobacteria phage HK022 (both Siphoviridae) (Figure 2c). These data are an indication that each phage genome has unique patterns of nucleotide usage that are not strictly determined by tail characteristics.

### Comparison of tetranucleotide usage patterns between bacteriophage genomes

**Figure 3:**
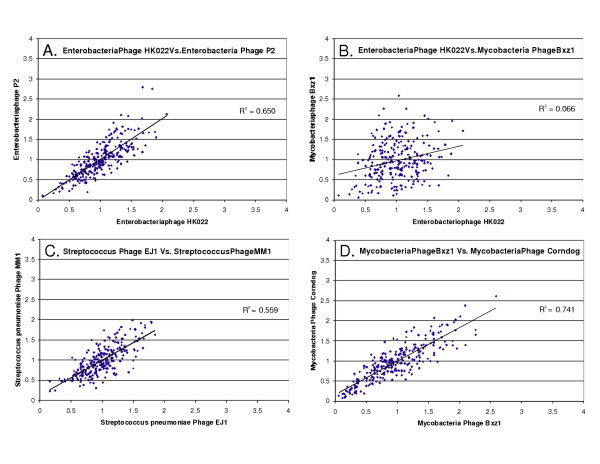
Linear regression analysis of DNA tetranucleotide usage profiles among selected genomes. Each of the 256 tetranucleotide combinations were determined for each genome as described in Materials and Methods, and the profiles compared by linear regression analysis. Panels A: Enterobacteria phage HK022 (Siphoviridae) vs. P2 (Myoviridae). Panel B: Enterobacteria phage HK022 vs. *Mycobacterium *phage Bxz1 (Myoviridae). Panel C: *Streptococcus pneumoniae *phage EJ1 (Myoviridae) vs. MM1 (Siphoviridae). Panel D: *Mycobacterium *phage Bxz1 vs. Corndog (Siphoviridae).

Comparison of TUD profiles by linear regression analysis reveals that phages with comparable host range have similar patterns. Enterobacteria phage HK022 is more closely related in TUD to Enterobacteria phage P2 (R^2 ^= 0.649; Figure 3a) than to *Mycobacterium *phage Bxz1 (R^2 ^= 0.067; Figure 3b). A parallel relationship is shown by comparing *Burkholderia cepaciae *phage 1 with *Burkholderia cepaciae *phage 781 (R^2 ^= 0.980), and with "closely related" *Bordetella *phage BIP-1 (R^2 ^= 0.561). The two Enterobacteria phages (Figure 3a) share high level TUD similarity despite different tail morphology; results are parallel for *Streptococcus *phages (R^2 ^= 0.559; Figure 3c) and for *Mycobacterium *phages (R^2 ^= 0.741; Figure 3d). These representative examples show that TUD patterns may not be predicted by tail morphology, suggesting that family classification in bacteriophages is not phylogenetically robust.

### Bacteriophage tetranucleotide phylogeny

**Figure 4:**
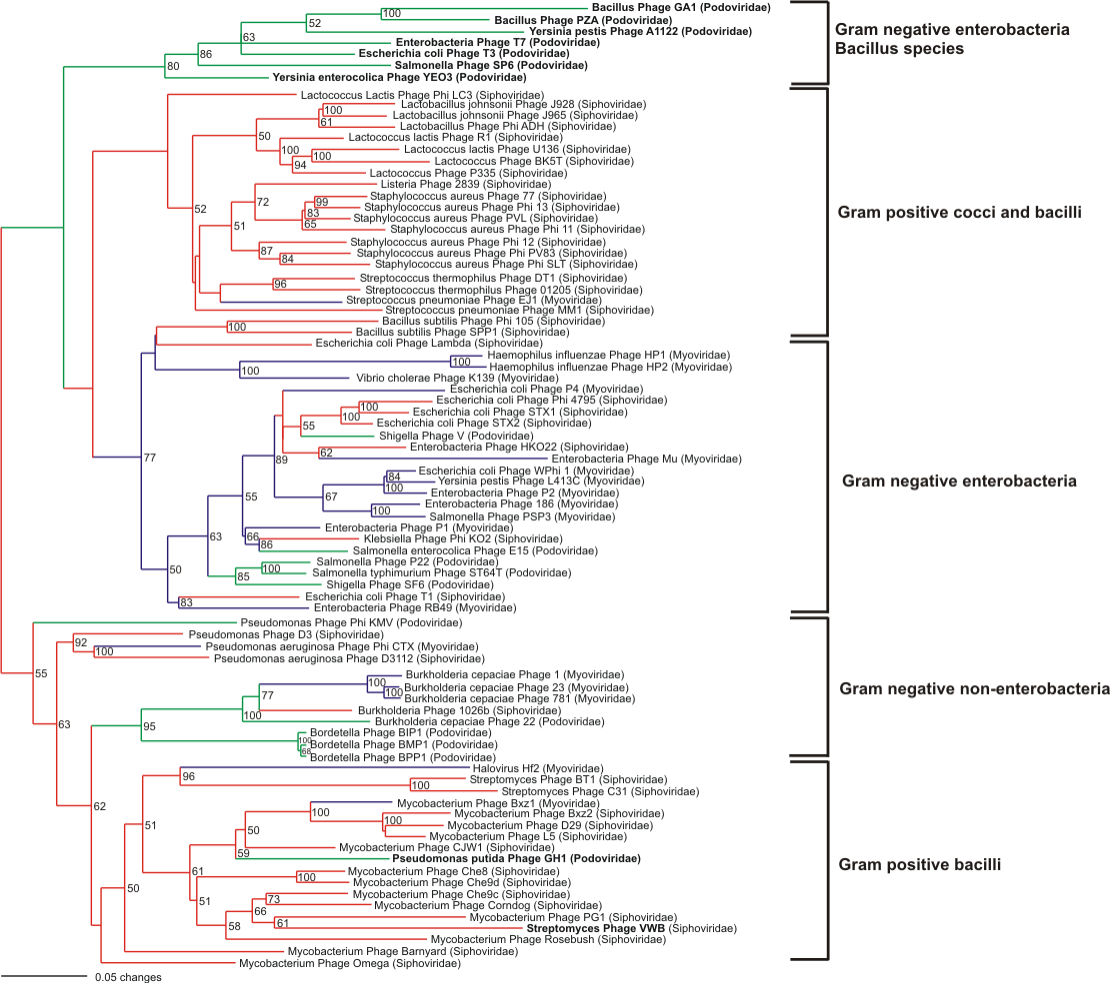
Phylogram of 83 selected bacteriophages for which genomic sequences are available. The organisms were grouped by using distance matrices based on the sums of the differences from the other organisms for the 256 tetranucleotide combinations, as described in Materials and Methods. Phylogenies were created by neighbor-joining analysis. Colors indicate Myoviridae (contractile tails; blue), Podoviridae (short tail stubs; green), and Siphoviridae (long tails; red). Bootstrap values >50 based on 100 replicates are represented at each node, and the branch length index is indicated below the phylogeny. Bacteriophage source by host species – gram-positive bacilli, gram-positive cocci, gram-negative enterobacteria, and gram-negative non-enterobacteria are indicated by brackets.

**Figure 5:**
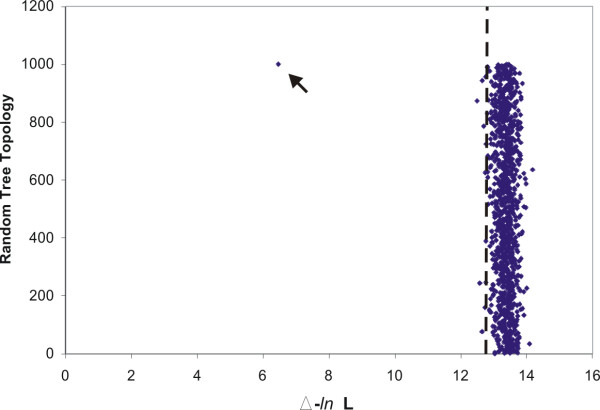
Likelihood analysis of phylogenetic congruence between the bacteriophage TUD phylogeny shown in Figure 4 and random trees. The 99^th ^percentile of the likelihood differences between the TUD tree and the topologies from 1000 random trees is indicated by the vertical dashed line. The position of the TUD phylogeny (indicated by the arrow) is substantially outside of the null distribution.

TUD patterns in prokaryotes have phylogenetic content similar to that of 16S rRNA [19]. Since our initial analysis indicated that TUD patterns are shared among bacteriophages with similar host range, we examined TUD-based phage phylogenetic structure to determine host-range influence on evolutionary relationships. Phylogenies based on TUD patterns were generated assuming that phylogenetic structure would be predicted by host range, tail morphology, a combination of both, or essentially be random. Compared with randomly generated phylogenies, TUD phylogenies (Figure 4) show a distinct, non-random pattern (Figure 5). With few exceptions, of the 83 test phages, TUD phylogenies have the following structure: 1) phages that parasitize gram-negative Enterobacteria (including *Escherichia*, *Shigella*, *Klebsiella*, *Yersinia*, *Salmonella*, and *Vibrio*) cluster together; 2) phages related to gram-positive cocci (including *Staphyloccus*, *Streptococcus*, and *Lactococcus *phages) cluster together, 3) phages related to gram-negative non-enterobacteria (including *Burkholderia*, *Pseudomonas*, and *Bordetella *phages) cluster together, and 4) phages related to gram-positive bacilli (including *Bacillus*, *Lactobacillus*, *Mycobacterium*, and *Streptomyces*) do not cluster. The observed clustering does not associate with tail morphology.

### Host-bacteriophage tetranucleotide phylogeny

**Figure 6:**
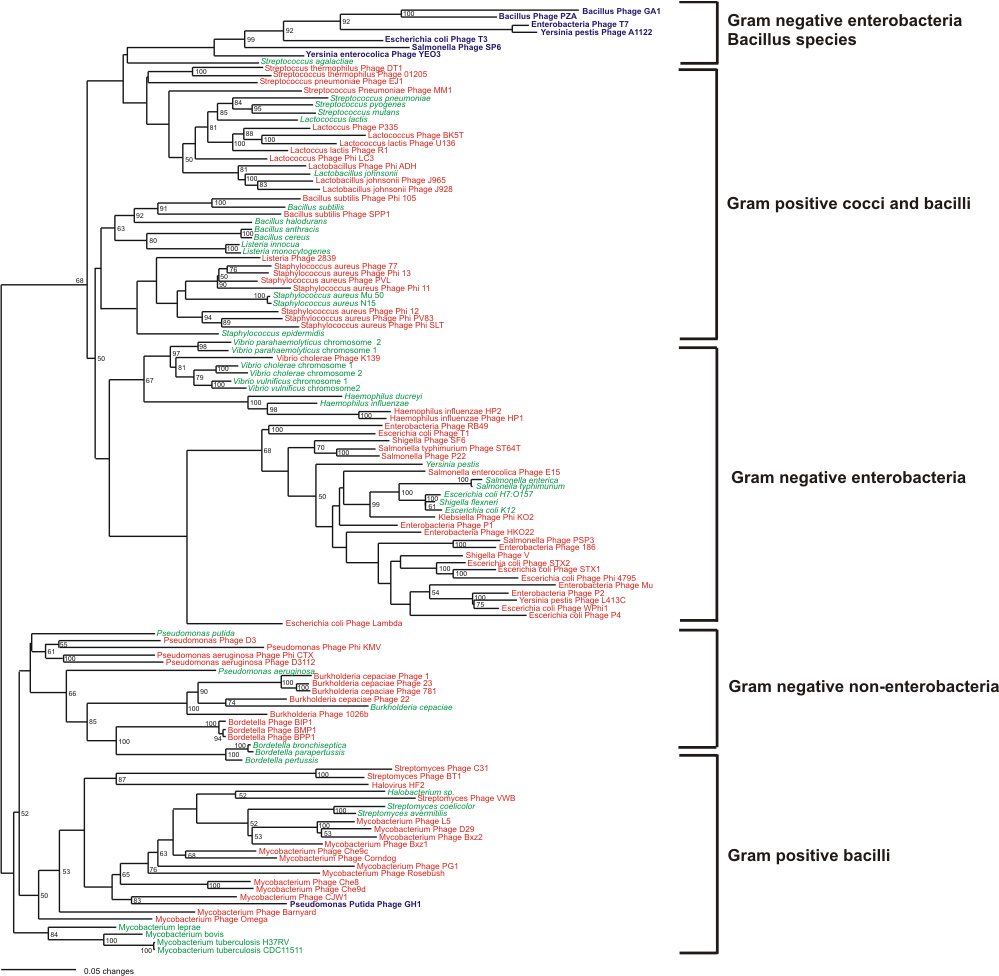
Phylogram of 39 selected bacteria and 83 selected bacteriophages for which genomic sequences are available. The organisms were grouped by using distance matrices based on the sums of the differences from the other organisms for the 256 tetranucleotide combinations, as described in Materials and Methods. Phylogenies were created by neighbor-joining analysis. Colors indicate bacteriophages (red), bacteria (green), and bacteriophages that are substantially beyond their presumed distribution (blue). Bootstrap values >50 based on 100 replicates are represented at each node, and branch length index is indicated below the phylogeny. Phages isolated in gram-positive bacilli, gram-positive cocci, gram-negative enterobacteria, and gram-negative non-enterobacteria are indicated by the brackets.

Since TUD-based phage phylogeny appears closely associated with host range, we determined the phylogenetic structure of bacteriophages together with their host bacteria. Nucleotide usage patterns in both bacteriophages and prokaryotes represent genomic signatures that can be compared across organisms in genome size-independent analysis [19,20]. With few exceptions, phages cluster near their host organisms, as is demonstrated by the close relationships with the Enterobacteria, *Staphylococcus*, *Streptococcus*, *Lactococcus*, *Lactobacillus*, *Listeria*, *Bordetella*, *Pseudomonas*, *Burkholderia*, *Streptomyces*, and *Mycobacteria *(Figure 6). The phylogeny is consistent with a model of host-phage co-evolution, but is inconsistent with phage evolution based on tail morphology. Exceptions to the model include *Bacillus *phages PZA and GA-1, *Pseudomonas *phage Phi KMV, and several Enterobacteria phages that cluster independent of presumed host (Figure 6).

### Eukaryotic virus TUD phylogeny

**Figure 7:**
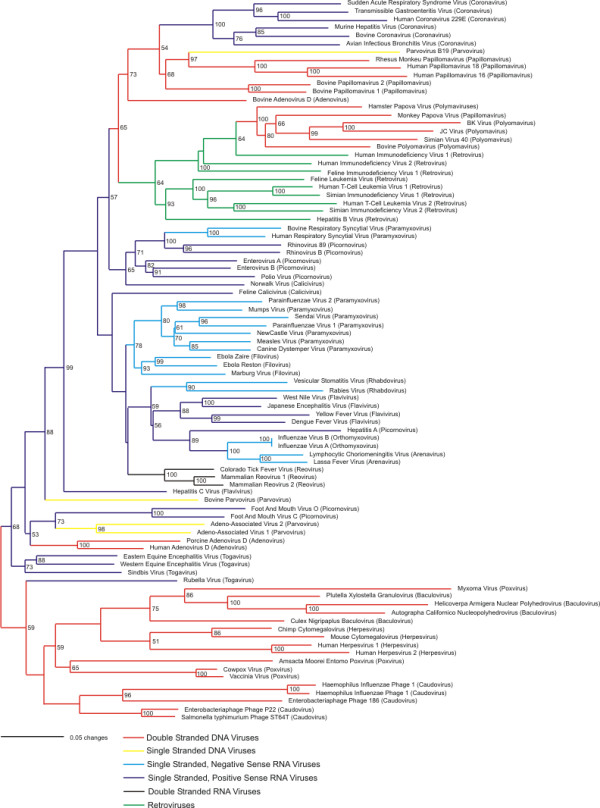
Phylogram of 90 selected viruses for which genomic sequences are available. The organisms were grouped by using distance matrices based on the sums differences from the other organisms for the 256 tetranucleotide combinations, as described in Materials and Methods. Phylogenies were created by neighbor-joining analysis. Colors indicate double stranded DNA viruses (red); single stranded DNA viruses (yellow); retroviruses (green); negative-sense single stranded RNA viruses (blue); positive-sense single stranded RNA viruses (sky blue); and double stranded RNA viruses (black). Bootstrap values >50 based on 100 replicates are represented at each node, and branch length index is indicated below the phylogeny.

Eukaryotic virus taxonomy also has been based on morphologic characteristics [2]. Since phage TUD phylogeny demonstrates substantial host influences (Figure 6), we determined eukaryotic virus TUD-based phylogeny using representative species to determine phylogenetic structure. Based on TUD patterns, eukaryotic viruses are associated based on family, size, and type of genetic material (Figure 7). Important trends include: 1) the single stranded RNA viruses cluster together, with the exception of togaviruses and coronaviruses; 2) within the RNA viruses, picornoviruses cluster with the exception of foot and mouth virus, and paramyxoviruses cluster with the exception of RSV; 3) segmented RNA viruses including orthomyxoviruses and arenaviruses cluster; 4) small double stranded DNA viruses cluster, but are separate from large double stranded DNA viruses; 5) polyomaviruses cluster with retroviruses in the group of small double stranded DNA viruses; 6) large double stranded DNA viruses, including bacteriophages cluster; and 7) single stranded DNA viruses including parvoviruses cluster variably. In summary, these data suggest that phylogenetic signal exists in the virus TUD patterns, but to variable extents in different virus groups.

### Gene content phylogeny of eukaryotic viruses

**Figure 8:**
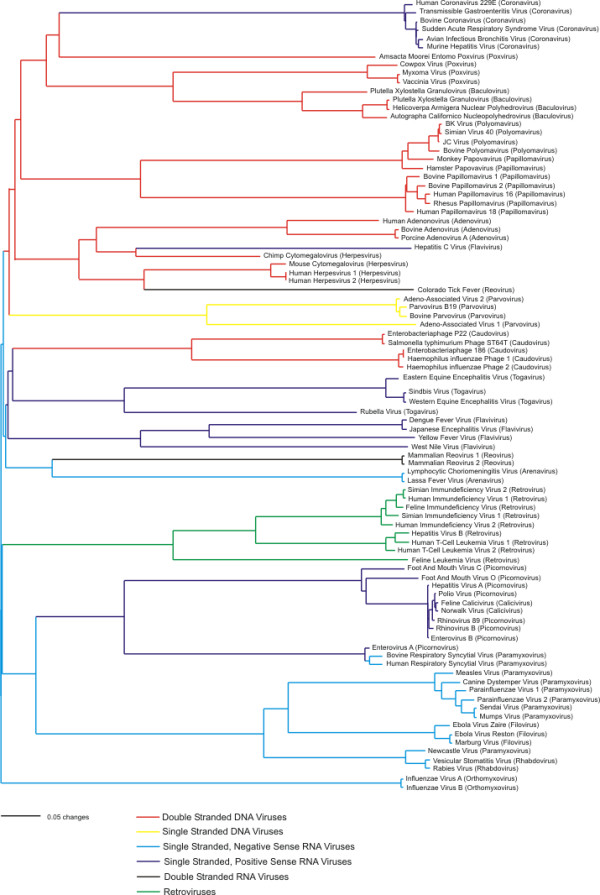
Phylograms of 90 selected viruses for which genomic sequences are available. The organisms were grouped according to distance matrices based on the number of shared orthologues between each genome, as described in Materials and Methods. Phylogenies were created by neighbor-joining analysis. Color code as is for Figure 7. The branch length index is indicated below the phylogeny.

For prokaryotes, phylogeny based on gene content also approximates that of 16S rRNA [17]. We determined the phylogeny of the representative eukaryotic viruses based on gene content as an independent measure of virus evolution, hypothesizing that closely related viruses share more gene content [16,26]. The eukaryotic viruses tested cluster in a pattern similar to those based on TUD (Figure 7), but with important differences (Figure 8). DNA viruses cluster on the top portion of the derived phylogeny, retroviruses share a more central position, and RNA viruses cluster on the lower portion with the exception of the coronaviruses. Phylogeny based on gene content appears more robust than TUD phylogeny for the positive-sense RNA viruses, both large and small DNA viruses, single stranded DNA viruses, and retroviruses, which cluster separately from the small DNA viruses.

## Discussion

As greater numbers of prokaryote and virus genomes are solved, genomic signatures have become better defined. Prokaryotes have genomic signatures that contain phylogenetic signal at both the dinucleotide [25] and tetranucleotide [19] levels. Our data indicate the existence of genomic signature in viruses parasitizing prokaryotic and eukaryotic hosts. Because genomic signature analysis is based on whole-genomes and is independent of multiple alignments, it provides a robust methodology for comparison across and between prokaryotes and viruses.

We show that bacteriophage genomic signatures are associated with their host organisms with few notable exceptions (Figure 6). This is most likely the effect of co-evolution between host and parasite, in which the host influences phage nucleotide usage patterns and possibly vice versa [25]. Indeed, both phage and host avoid use of certain tetranucleotides recognized by host restriction/modification systems [19]. The close approximation, but not complete identity of phage TUD patterns to those of host organisms supports a co-evolution model (Figure 6). We hypothesize that the differences reflect limitations in the ability of phages to adapt to host TUD patterns, and/or that phages need to retain particular TUD patterns to maintain host range.

An alternative explanation for bacteriophage TUD patterns is that their approximation to their hosts results from amelioration [27], and does not reflect recent common ancestry. Obligate parasitism likely necessitates that phages ameliorate to host nucleotide usage patterns, which is why host range may be critical. Bacteriophage phylogenetics based on shared gene content is dissimilar to that based on TUD, but not consistent with taxonomy based on morphologic features [26]. The differences between the methods likely reflect biases, with TUD phylogeny biased by amelioration, and gene content phylogeny biased by lateral gene transfer.

Horizontal gene transfer from host to phage produces apparent similarity to host nucleotide usage patterns that mechanistically does not represent recent common ancestry [28]. If horizontal transfer from host to phage served as the predominant mechanism of phage evolution, *Mycobacterium *phage TUD patterns would closely reflect their hosts. In the *Mycobacterium *phages, where horizontal acquisition is common [7], phage TUD patterns more closely approximate each other than their prokaryote hosts (Figure 6), which supports host-phage evolution in parallel, but not their evolution through horizontal gene transfer. That nucleotide usage patterns are relatively homogenous across each phage genome (Figure 1), suggests that the proportion of horizontally transferred genes in phage genomes may be relatively small or subject to rapid amelioration, and substantiates a parallel model of host-phage evolution. In phages with known transposons such as Enterobacteria phage Mu, these elements are not identified as anomalous in the phage genome (Figure 1c), suggesting that transposons are not responsible for the observed similarity in nucleotide usage patterns between host and phage.

Our data on bacteriophage phylogenies (Figure 5) directly oppose conceiving of morphological characteristics for understanding phage ancestry. Based on a TUD-based model of evolution, tail characteristics would have been horizontally transferred, subject to variable rates of evolution, or be continuously altered with little phylogenetic significance. Conversely, for a model of ancestry based on tail morphology to be correct, patterns of nucleotide usage would have to had shifted continuously throughout phage evolution independent of host. The substantial relationships between phage and host TUD (Figure 6) make independent evolution highly unlikely. Exceptions to the model of host-phage co-evolution presented by Podoviruses *Bacillus *phages PZA and GA-1, *Pseudomonas putida *phage GH-1, and a few Enterobacteria phages could be explained by broad host range, accelerated rates of change with loss of TUD phylogenetic signal, or alternate replication strategies. Each of these Enterobacteria phages is T7-like, with many known similarities at the genetic level [29], further suggesting their phylogenetic distinctness. The Bacillus phages are Phi29-like phages, which in addition to lack of shared ancestry with other Bacillus phages (Figure 4), have T7-like polymerases [30], supporting their recent ancestry with T7-like phages. Each of these phage groups has substantial strand bias [31], indicating their unique differences compared to other phage groups, and likely evolutionary distance.

TUD phylogenetics support co-evolution of host and bacteriophage, however, eukaryotic viruses cluster similar as expected based on recognized genetic and morphological features [1,2]. Bovine viruses (e.g. bovine RSV, papillomavirus, coronavirus, parvovirus, and polyomavirus) cluster independent of host, indicating that factors other than host influences determine their phylogenetic position (Figure 7). That coronaviruses do not belong to the major RNA virus cluster (Figures 7 and 8), contrary to previous studies [32], suggests that TUD may not be robust for certain RNA viruses. Most of the negative-sense RNA viruses cluster with the exception of RSV and the segmented RNA viruses. The clustering of RSV with rhinoviruses may represent convergent evolution or allelic exchange, as they occupy a similar ecological niche. The phylogenetic position of the segmented RNA viruses including orthomyxoviruses, arenaviruses, and reoviruses supports the concept of a common progenitor (Figure 7). The TUD analysis shows separate grouping of the large and small double stranded DNA viruses, which may be a limitation of the technique or reflect the occurrence of double stranded DNA more than once in the ancestral history of viruses. Bacteriophages cluster with the eukaryotic double stranded DNA viruses, further suggesting a single progenitor for the large double stranded DNA viruses. Whether the polyphylogeny observed among the large and small DNA viruses, the positive-sense RNA viruses, and the single stranded DNA viruses reflect methodologic limitations or evolutionarily significant phenomena remains to be determined. Phylogeny based on gene content, in which polyphylogeny among each group of viruses is diminished, supports the former hypothesis (Figure 8).

## Conclusion

Morphological features form one basis for virus taxonomy, however we provide data that suggests bacteriophage tail characteristics may not sufficiently reflect their evolution. Based on TUD patterns, phages are co-evolving with their hosts in a manner defined by their ability to achieve broad host range. That there are only few exceptions to the co-evolution model concerning the many phages analyzed, substantiates that phylogenetic signal exists in phage TUD patterns. The TUD methodology is easily reproducible, alignment-independent, affected by lateral gene transfer in proportion to the extents of transfer, and can be used for directly comparing both prokaryotes and viruses. Despite differences between TUD and morphology based classification, TUD phylogeny retains utility in understanding host-phage co-evolution and deviation in patterns of nucleotide usage in certain viruses since their divergence from recent common ancestors. As such, TUD phylogeny should be considered complementary to other systems for analysis of virus evolution.

## Methods

### Virus, phage, and microbial genomes

Complete genome sequences of the phages [see Additional file 1], viruses [see Additional file 2], and bacteria studied were obtained from Genbank [33,34].

### Analysis of tetranucleotides

Tetranucleotides were selected for study because analysis of higher-order oligonucleotides was not possible given the limitation in virus genome sizes. We based our minimum genome sequence length on the assumption that 95% of tetranucleotide combinations should occur at least 10 times [31]. Our calculated minimum length was 5 kb based on analysis of concatenated genome strands designed to eliminate strand bias. The minimum genome length analyzed in this study was 4.7 kb (9.4 kb when analyzing both strands), while analysis of pentanucleotides would have required a minimum genome length of 20 kb. To determine the tetranucleotide usage deviations from expected (TUD) among prokaryotic genomes, a Zero-Order Markov algorithm [35] was used, which involves determining the expected number of tetranucleotides by removing biases in mononucleotide frequencies, as is determined by the equation: *E(W) *= [(A^a ^* C^c ^* G^g ^* T^t^) * N], where A, C, G, and T represent the frequency of the four nucleotides within the window being evaluated, respectively, a, c, g, and t represent the number of nucleotides A, C, G, and T in each tetranucleotide, respectively, and N represents the length of the genome being evaluated. The frequency of divergence for each tetranucleotide is expressed as the ratio of observed to expected, and the TUD profiles for all tetranucleotides determined for each organism studied using Swaap 1.0.1 [36], and their relative abundance between genomes compared by linear regression analysis.

### Tetranucleotide difference index

Tetranucleotide differences in each genome were determined using Markov chain analysis [37], by determining the expected oligonucleotide word frequency by removing the biases existing in component oligonucleotides. *W *= (*w*_*1*_*w*_*2*_...*w*_*m*_) denotes the word formed by the concatenation of *m *nucleotides, and *N(W) *is its observed count in a sequence of length *n*, as described [24]. The expected count *E(W) *of *W *is:

*E(W) *= *N(w*_*1*_*w*_*2*_...*w*_*m*-*1*_*)N(w*_*2*_*w*_*3*_...*w*_*m*_*)/N(w*_*2*_*w*_*3*_...*w*_*m*-*1*_*)*.

The frequency of the word *F(W) *is expressed as the ratio of the observed *O(W) *to the expected *E(W)*. *F(W) *can be determined for all windows *F*_*w*_*(W) *of specified size within each genome. Tetranucleotide differences for each window are measured by the expression: ∑ji|F(W)−Fw(W)|
 MathType@MTEF@5@5@+=feaafiart1ev1aaatCvAUfKttLearuWrP9MDH5MBPbIqV92AaeXatLxBI9gBaebbnrfifHhDYfgasaacH8akY=wiFfYdH8Gipec8Eeeu0xXdbba9frFj0=OqFfea0dXdd9vqai=hGuQ8kuc9pgc9s8qqaq=dirpe0xb9q8qiLsFr0=vr0=vr0dc8meaabaqaciaacaGaaeqabaqabeGadaaakeaadaaeWaqaamaaemaabaacbiGae8NrayecbaGae4hkaGIae83vaCLae4xkaKIaeyOeI0Iae8Nray0aaSbaaSqaaiab=Dha3bqabaGccqGFOaakcqWFxbWvcqGFPaqkaiaawEa7caGLiWoaaSqaaiab+PgaQbqaaiab+LgaPbqdcqGHris5aaaa@3F04@, where i - j represent all tetranucleotide combinations. The tetranucleotide difference index represents the difference between each window and the mean difference for all windows. Z-scores were determined for each window using the equation Z = (x - μ)/σ, where x represents the tetranucleotide difference for the window being evaluated, μ represents the mean tetranucleotide differences for all windows, and σ represents the standard deviation of all windows. Those windows differing from the mean by ± 3 Z-scores were defined as significant, and were determined using Swaap 1.0.1 [36].

### Phylogenetic analysis

Distances based on TUD were determined: D_t _= 1/4^4 ^* ∑ |*F*_*1*_*(W) *- *F*_*2*_*(W)*|, where *F*_*1*_*(W)*and *F*_*2*_*(W) *represent *F(W) *for each of the 256 tetranucleotides for any organisms 1 and 2 [19,38], which represents the Euclidean distance between 2 vectors in 256 space. Bootstraping was performed by sampling with replacement of each of the 256 tetranucleotide frequencies using Swaap PH 1.0.1 [39], and phylograms created based on distance matrices using Phylip 3.5 [40], reviewed via Treeview [41], and displayed using midpoint rooting with Paup 4.0b10 [42].

Phylogeny based on gene content was determined using methodology similar to that described [17]. Shared genes between genomes were determined using an operational definition of orthology. Briefly, all genes within each genome examined were added to a BLAST database, and each genome then was compared at the amino acid level against the database using a BLAST threshold value, E = 0.01. The resulting number of orthologues were tabulated for each pair of viruses compared, and distances were expressed as one minus the percent of shared genes between each genome. Data tabulation and distance matrices were generated using Swaap PH 1.0.1 [39]. Phylogenies were constructed based on distance matrices using Paup 4.0b10 [42].

### Analysis of congruence among phylogenetic trees

Analysis of congruence among the gene phylograms was performed on consensus trees, and 1000 trees created with random topology. Differences in log likelihood (Δ-*ln *L) were computed between phylograms based on TUD phylogenies and 1000 random trees. Differences in Δ-*ln *L for random phylograms can be considered as the null distribution, obtained when there is no more similarity in topology than expected by chance. If the Δ-*ln *L values for comparisons among the phylograms are within the 99^th ^percentile of the null distribution, then the topologies are significantly different, and thus incongruent [43].

## List of Abbreviations

TUD – Tetranucleotide usage deviations from expected

## Authors' contributions

DP – conceived of study, carried out genome analysis, revised software, and drafted manuscript

TW – participated in study design, data analysis, and manuscript revision

CG – participated in study design, literature review, data analysis, and manuscript revision

MB – participated in study design and manuscript revision

All authors read and approved the final manuscript
